# Characterization of *Caleosin* Genes in *Carica papaya* and Insights into Lineage-Specific Family Evolution in Brassicales

**DOI:** 10.3390/plants14213296

**Published:** 2025-10-29

**Authors:** Zhi Zou, Xiaowen Fu, Xiaoping Yi, Chunqiang Li, Yongguo Zhao

**Affiliations:** 1 National Key Laboratory for Tropical Crop Breeding/Hainan Key Laboratory for Biosafety Monitoring and Molecular Breeding in Off-Season Reproduction Regions, Institute of Tropical Biosciences and Biotechnology/Sanya Research Institute of Chinese Academy of Tropical Agricultural Sciences, Haikou 571101, China; 2 College of Biology and Food Engineering, Guangdong University of Petrochemical Technology, Maoming 525000, China

**Keywords:** caleosin, peroxygenase, phylogenomics, orthologous analysis, syntenic analysis, evolution pattern

## Abstract

Caleosins (CLOs) or peroxygenases (PXGs), a class of structural proteins of lipid droplets (LDs), comprise a small family of multifunctional proteins widely involved in oil accumulation, organ development, and stress responses. Despite the proposal of two clades termed H and L in *Arabidopsis thaliana*, their evolution in the order Brassicales has not been well established. In this study, the first genome-wide analysis of the *caleosin* family was conducted in papaya (*Carica papaya*), a Caricaceae plant without any recent whole-genome duplication (WGD). A high number of five members representing both H and L clades were identified from the papaya genome. Further identification and comparison of 68 *caleosin* genes from 14 representative plant species revealed seven orthogroups, i.e., H1–4 and L1–3, where H1 and L1 have already appeared in the basal angiosperm *Amborella trichopoda*, supporting their early divergence before angiosperm radiation. Five *CpCLO* genes belong to H1 (1) and L1 (4), and extensive expansion of the L1 group was shown to be contributed to by species-specific tandem and transposed duplications, which may contribute to environmental adaptation. Orthologous and syntenic analyses uncovered that lineage-specific expansion of the *caleosin* family in Brassicales relative to *A. trichopoda* was largely contributed to by tandem duplication and recent WGDs, as well as the ancient γ whole-genome triplication (WGT) shared by all core eudicots. Independent gain or loss of certain introns and apparent expression divergence of *caleosin* genes were also observed. Tissue-specific expression analysis showed that *CpCLO2* and −*5* are constitutively expressed, whereas others appear to be tissue-specific, implying function divergence. Interestingly, the H-forms *CpCLO1* and *RcCLO1* were shown to exhibit similar expression profiles to most *oleosin* genes that are preferentially expressed oil-rich tissues such as seeds/endosperms, shoots, and calluses, implying their putative involvement in oil accumulation, as observed in *Arabidopsis*. The results obtained from this study provide a global view of *CpCLO* genes and insights into lineage-specific family evolution in Brassicales, which facilitates further functional studies in papaya and other non-model species.

## 1. Introduction

Caleosins (CLOs)/peroxygenases (PXGs) comprise a small family of multifunctional proteins that are involved not only in the formation, stabilization, and degradation of lipid droplets (LDs), but also in a wide range of plant development and stress responses [1,2,3,4]. Though they were first identified as a class of minor LD proteins in sesame (*Sesamum indicum*) seeds [5], other intracellular locations have also been reported, e.g., endoplasmic reticulum (ER), vacuole, plasmalemma, and chloroplast envelope fractions [6,7,8]. Additionally, they are also abundant in several non-seed tissues, e.g., megagametophytes, pollen, fruits, leaves, stems, and roots [6,9,10,11,12,13,14,15]. Structurally, caleosins exhibit some similarities to oleosins (OLEs), the major LD proteins that include three main domains, i.e., an N-terminal hydrophilic domain, a central hydrophobic domain, and a C-terminal hydrophilic domain [2,16,17]. Compared with oleosins, the central hydrophobic domain of caleosins is relatively shorter (i.e., 20 vs. 72 residues) and usually features a proline-knot pattern of PX_3_PSX_3_P (X represents a nonpolar residue) that is somewhat different from PX_5_SPX_3_P, as observed in oleosins [16,18,19,20,21,22,23]. Moreover, the N-terminal hydrophilic domain of caleosins typically possesses a single EF-hand calcium binding motif, whereas the C-terminal hydrophilic domain usually harbors several kinase phosphorylation sites [1,2,16,24,25]. Ca^2+^ is essential for their peroxygenase activity, which is dependent on a heme group coordinated by the two invariant His residues located in N- and C-terminal regions [16,24,25,26]. The distribution of caleosins is also wider, which are present not only in land plants but also in some fungi and green algae [27,28,29,30].

Genome-wide surveys showed that the *caleosin* family in land plants is highly diverse, varying from two to 22 members [18,19,27,28,29,30,31,32]. Interestingly, two members were not only reported in the basal angiosperm *Amborella trichopoda*, but also in three core eudicots, i.e., castor bean (*Ricinus communis*), flax (*Linum usitatissimum*), and cucumber (*Cucumis sativus*), in stark contrast to eight as described in the model plant *Arabidopsis thaliana* [18,19,30]. According to phylogenetic analysis, these caleosins could be divided into two main clades, i.e., H and L, with high and low molecular weights (MWs), respectively, where the L clade was supposed to evolve from the H clade due to the fragment deletion at the N-terminal [19,32]. The presence of both H- and L-forms in *A. trichopoda* implies early divergence of these two clades before angiosperm radiation. It has been established that after the split with *A. trichopoda*, the last common ancestor of core eudicots (including castor bean, flax, cucumber, and *A. thaliana*) underwent one so-called γ whole-genome triplication (WGT), whereas *A. thaliana* further experienced two lineage-specific whole-genome duplications (WGDs) known as β and α [33,34]. *A. thaliana* was shown to harbor four H-forms (i.e., *AtCLO1*, −*2*, −*3*, and −*8*) and four L-forms (i.e., *AtCLO4*−*7*), where two pairs (i.e., *AtCLO4*/−*6* and *AtCLO5*/−*7*) are organized in tandem repeats and another two pairs (i.e., *AtCLO1*/−*2* and *AtCLO5*/−*6*) were characterized as WGD repeats [19]. Nevertheless, the exact mechanisms contributing to the extensive expansion of the *AtCLO* family and lineage-specific evolution in Brassicales are yet to be studied.

Papaya (*Carica papaya* L.) belongs to the Caricaceae family within the order Brassicales, which includes 17 families and approximately 400 genera [35]. Though multiple independent recent WGDs have been described in Brassicales [36], no additional WGD after the γ event was detected in papaya, which was estimated to have diverged with *A. thaliana* about 72 million years ago (Mya) [37]. From an evolutionary perspective, analyzing papaya genes could provide insights into lineage-specific family evolution in this economically and ecologically important plant order [23,38,39]. In this study, a systemic analysis of *caleosin* genes was conducted in papaya, including sequence characteristics, gene structures, chromosomal localizations, evolutionary relationships, and expression profiles, as well as a comprehensive comparison with representative plant species such as *A. thaliana*, castor bean, *Aquilegia coerulea*, and *A. trichopoda*. Significantly, 1:1 orthologous relationships were observed between castor bean and *A. trichopoda*, whereas 1:1 and 4:1 were found between papaya and castor bean/*A. trichopoda*. Moreover, our results showed that the expansion of the *CpCLO* family was contributed by tandem, transposed, and dispersed duplications, in contrast to a more important role of WGDs in Brassicaceae plants. Apparent expression divergence of *caleosin* genes was also observed, implying putative function divergence. Herein, we report our findings.

## 2. Results

### 2.1. Identification, Chromosomal Localization, and Duplication Event Analysis of Five Caleosin Genes in Papaya

**Figure 1:**
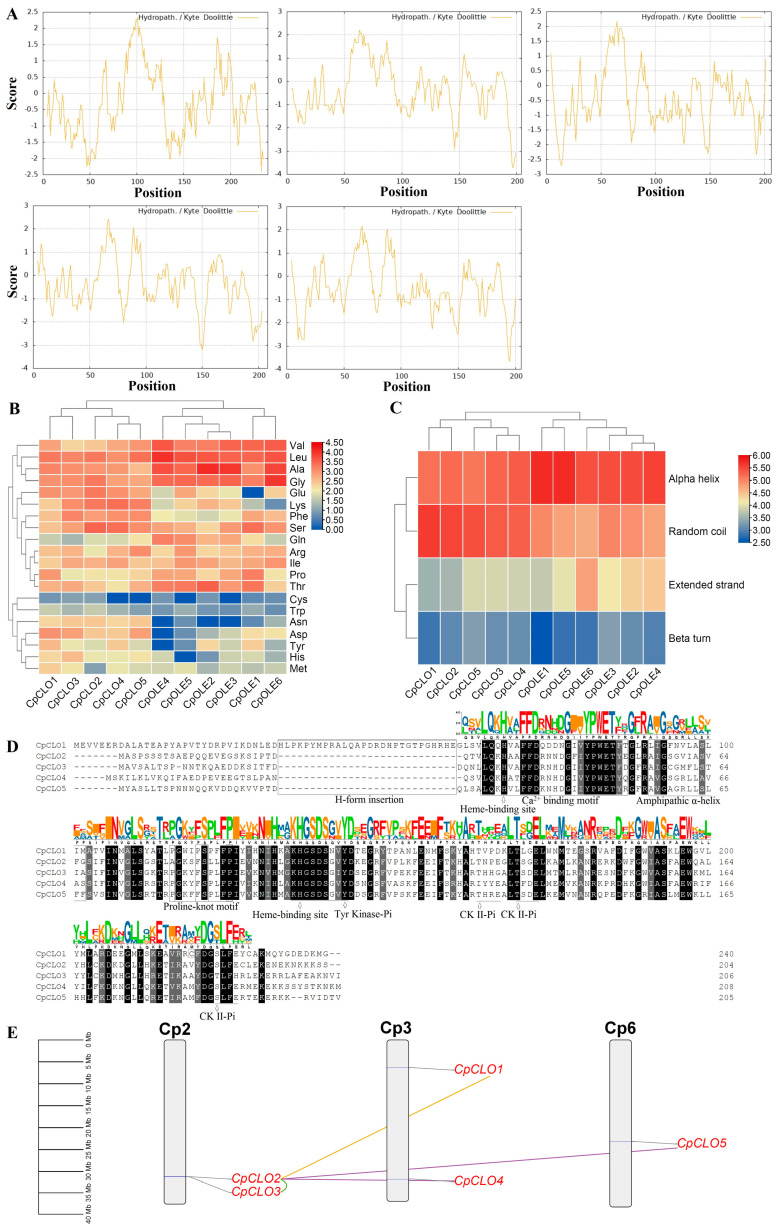
Sequence and duplication event analyses of *caleosin* family genes in *C. papaya*. (**A**) Kyte–Doolittle hydrophobicity plots of five CpCLOs analyzed using ProtScale. (**B**) Comparison of amino acid composition between CpCLOs and CpOLEs, which was calculated using ProtParam. (**C**) Comparison of secondary structure between CpCLOs and CpOLEs, which was identified using the SOPMA method. (**D**) Multiple sequence alignment of CpCLOs performed using MUSCLE. Broken lines in the sequences represent gaps introduced for best alignment, whereas identical and similar amino acids are highlighted in black and dark gray, respectively. The SeqLogo of the caleosin domain generated using WebLogo is shown above the alignment, and the positions of the H-form insertion, an EF-hand calcium binding motif, the amphipathic α-helix, and the proline knot-like motif (PX_3_P/FSX_3_P) are boxed in black and are indicated at the bottom of the sequences. Two invariable His residues for heme-binding and four phosphorylation sites (one for Tyr kinase and three for CK II) are underlined and pointed to by arrows. The Cys residue prior to the last CK II phosphorylation site is boxed in purple. (**E**) Chromosome localization and duplication events detected in papaya. Chromosome localization was conducted using TBtools-II, whereas different modes of gene duplication were identified using the DupGen_finder pipeline. Serial numbers are indicated at the top of each chromosome, and the scale is in Mb. Duplicate pairs identified in this study are connected using lines in different colors, i.e., tandem (green), transposed (purple), and dispersed (gold). (Cp: *C. papaya*; CK II: casein kinase II; CLO: caleosin; Mb: megabase; OLE: oleosin).

The search of the papaya genome resulted in five *caleosin* genes, which are slightly less than the six members as reported for the *oleosin* family [23]. Without any exception, all deduced CpCLO proteins were shown to harbor a single caleosin domain (under the Pfam accession number of PF05042) (Table 1). The peptide size varies from 204 (CpCLO2) to 240 (CpCLO1) residues, and the average length of 213 residues is relatively longer than 153 residues as observed for six CpOLEs [23]. Correspondingly, a higher MW with an average of 24.21 kilodalton (kDa) was found for CpCLOs relative to CpOLEs (16.10 kDa). The isoelectric point (pI) of CpCLOs varies from 5.05 (CpCLO1) to 9.57 (CpCLO5), where 60.00% of them (CpCLO1–3) have a pI value of less than 7, in contrast to only CpOLE6 harboring an acidic pI [23]. Interestingly, all CpCLOs possess a grand average of hydropathicity (GRAVY) value of less than 0, varying from −0.329 to −0.509, in stark contrast to the highly hydrophobic feature of CpOLEs [23]. Correspondingly, the ProtScale analysis showed that all CpCLOs exhibit similar hydropathicity scales and only the central region (more precisely the amphipathic α-helix and the proline-knot motif, see below) is hydrophilic (Figure 1A). Distinct amino acid (AA) composition and secondary structure were also observed between CpCLOs and CpOLEs. As shown in Figure 1B, except for the absence of the Cys in CpCLO4 and –5, other members contain all 20 residues, rich in Leu, Gly, Ala, and Val, as observed in CpOLEs. Compared with CpOLEs, CpCLOs usually contain more Glu, Lys, Phe, Ser, Asp, Asn, and Tyr, but less Leu, Gly, Ala, and Val, Gln, Thr, and Pro; they always harbor more hydrophilic but less hydrophobic residues and they also have more random coils but less alpha helixes and extended strands. Interestingly, the cluster analyses of amino acid and secondary structure percentages revealed that these proteins were grouped in families, though subgroups were also observed within each family (Figure 1B,C).

Sequence alignment of five CpCLOs is shown in Figure 1D. In contrast to the conservation of the caleosin domain, N- and C-terminal regions are highly diverse. CpCLO1 differs from other members with a 29-residue insertion, which was defined as the H insertion in *A. thaliana* [19]. This insertion mainly contributes to the longer peptide size and higher MW in CpCLO1 (Table 1). The caleosin domain is composed of a Ca^2+^ binding motif, an amphipathic α-helix, a proline-knot motif, two heme-binding sites, and four main phosphorylation sites, i.e., one for Tyr kinase and three for casein kinase II (CK II) (Figure 1D). The identified proline-knot pattern P/AX_3_P/FSX_3_P is somewhat different from PX_5_SPX_3_P, found in CpOLEs [23]. The heme-binding sites are composed of two invariable His residues, which were shown to be essential for peroxygenase activity [24], implying that they have similar functions. The first CK II phosphorylation site is usually a Thr residue, but it is placed by an Ile residue in CpCLO4, implying its divergence. Despite the presence of two Cys residues in CpCLO1–3, their positions are usually different, and the one just before the third CK II phosphorylation site is only found in CpCLO1 (Figure 1D).

Chromosome localization indicates that five *CpCLO* genes are unevenly distributed over three chromosomes (Chrs). Whereas *CpCLO5* is located on Chr6, *CpCLO1*/–*4* and *CpCLO2*/–*3* are co-located on Chr3 and Chr2, respectively (Figure 1E). Among them, *CpCLO2* and –*3* were defined as tandem repeats for their neighboring locations, where *CpCLO2* was characterized as the dispersed repeat of *CpCLO1*. Moreover, both *CpCLO4* and –*5* were characterized as transposed repeats of *CpCLO2*. A similar role of tandem and transposed duplications on the expansion of the *caleosin* family was also observed in *A. thaliana*, where *AtCLO4*/–*6* and *AtCLO5*/–*7* were characterized as tandem repeats and *AtCLO3*/–*8* were characterized as transposed repeats. The main difference is that *A. thaliana* harbors two α WGD repeats, i.e., *AtCLO1*/–*2* and *AtCLO5*/–*6* (Figure S1), which is consistent with a previous study [19].

The evolutionary rates of four duplicate pairs identified in the *CpCLO* family are summarized in Table 2. In accordance with early divergence of *CpCLO1* and –*2*, their Ks (synonymous substitution rate) value was not calculated due to high sequence divergence, whereas others vary from 0.8983 to 1.2871. The relatively higher Ks value observed between *CpCLO2* and –*4* implies the early origin of *CpCLO4* compared to *CpCLO3* and –*5*. Since the Ka (nonsynonymous substitution rate)/Ks values of all duplicate pairs are less than one, purifying selection may play a key role in their evolution.

### 2.2. Comparison of Caleosin Genes in Papaya and A. thaliana Revealed Species-Specific Evolution

**Figure 2:**
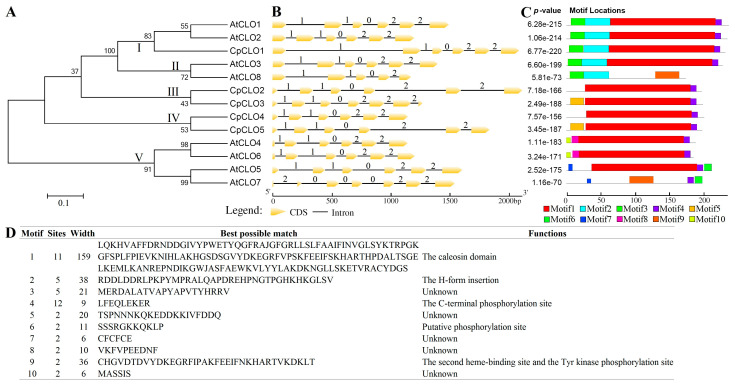
Structural and phylogenetic analyses of *caleosin* family genes in *C. papaya* and *A. thaliana*. (**A**) Shown is an unrooted phylogenetic tree resulting from full-length caleosins with RAxML (maximum likelihood method and bootstrap of 1000 replicates), where the distance scale denotes the number of amino acid substitutions per site and the name of each clade is indicated next to the corresponding group. (**B**) Shown are the exon–intron structures. “0”, “1”, and “2” indicate that an intron is located between codons, the first and second bases of a codon, and the second and third bases of a codon, respectively. (**C**) Shown is the distribution of conserved motifs identified using MEME, where different motifs are represented by different color blocks as indicated and the same color block in different proteins indicates a certain motif. (At: *A. thaliana*; Cp: *C. papaya*; CLO: caleosin). (**D**) Shown is the detailed information of ten motifs identified in this study.

Despite residing in the same order of Brassicales, there are five *CpCLO* genes identified in papaya, which is relatively less than the eight present in *A. thaliana* [19], reflecting the occurrence of two additional WGDs in the latter [33]. To uncover their evolutionary relationships, an unrooted phylogenetic tree was constructed using full-length peptides. As shown in Figure 2A, 13 caleosins were clustered into five groups: Group I includes three members from two species, i.e., CpCLO1, AtCLO1, and AtCLO2; Group II and V are *A. thaliana*-specific, including AtCLO3/–8 and AtCLO4/–5/–6/–7, respectively; and Group III and IV are papaya-specific, including CpCLO2/–3 and CpCLO4/–5, respectively. Among them, Groups I and II belong to the H clade, as described in *A. thaliana* [19], where CpCLO1 exhibits 84.21% and 83.33% sequence similarities with AtCLO1 and AtCLO2, which are relatively higher than the similarities of 76.86% and 57.68% with AtCLO3 and AtCLO8, respectively. Groups III, IV, and V belong to the L clade, where 68.37–74.76% sequence similarities were observed among CpCLO2–5, usually higher than those of among AtCLO4–7 (Table S1). Interestingly, comparative sequence similarities of 70.33–74.04% were observed between AtCLO4 and CpCLO2–5 (Table S1), implying their close relationships.

Exon–intron structures of *Cp*/*AtCLO* genes were further investigated. As shown in Figure 2B, most genes feature five introns with the phase pattern of 1, 1, 0, 2, and 2, where “0”, “1”, and “2” indicate that an intron is located between codons, the first and second bases of a codon, and the second and third bases of a codon, respectively. Nevertheless, *CpCLO3* was shown to have gained one more intron in phase 2 at the 3′ end; *AtCLO8* has lost the last intron and exhibits the phases 1, 1, 0, and 2; and *AtCLO7* harbors the unusual intron phases of 2, 0, 0, 0, 2, and 2, implying their divergence.

The conserved motifs of Cp/AtCLOs were also compared. Among ten motifs identified using MEME, Motifs 1 and 4 are widely distributed (Figure 2C,D), which were characterized as the caleosin domain and the C-terminal phosphorylation site, as shown in Figure 1D, respectively. By contrast, other motifs appear to be group or sequence-specific, i.e., Motifs 2 and 3 for H-forms, Motif 9 for AtCLO7 and –8, Motif 5 for CpCLO3 and –5, Motifs 6 and 7 for AtCLO5 and –7, Motifs 8 and 10 for AtCLO4 and –6 (Figure 2C). Among them, Motif 2 was characterized as the H-form insertion (Figure 2D), whereas Motif 9 was also characterized as the caleosin domain, more precisely the region including the second heme-binding site and the Tyr kinase phosphorylation site, as shown in Figure 1D. These results support the divergence of H- and L-forms and imply possible functional divergence even within the five groups identified in this study.

### 2.3. Characterization of Caleosin Genes from Representative Plant Species and Insights into Lineage-Specific Family Evolution in Brassicales

Distinct evolution patterns observed between papaya and *A. thaliana* impelled us to investigate whether the identified duplication events were species or lineage-specific. For the purpose, *caleosin* family genes were further identified from representative plant species, including *A. trichopoda* (the basal angiosperm in Amborellales), *Aquilegia coerulea* (a Ranunculales member of the basal-most eudicot clade that did not share the γ WGT), castor bean (an Euphorbiaceae plant in Malpighiales that did not experience any recent WGD), and nine Brassicales plants, i.e., horseradish tree (*Moringa oleifera*, a Moringaceae plant that did not experience any recent WGD), *Bretschneidera sinensis* (an Akaniaceae plant that experienced one recent WGD known as Bs-α), caperbush (*Capparis spinosa*, a Capparaceae plant that experienced the β WGD and one so-called Cs-α WGD), *Cleome violacea* (a Cleomaceae plant that experienced the β WGD), acaya (*Gynandropsis gynandra*, a Cleomaceae plant that experienced the β WGD and one so-called Gg-α WGD), spider flower (*Tarenaya hassleriana*, a Cleomaceae plant that experienced the β WGD, the Gg-α WGD, and one so-called Th-α WGD), saltwater cress (*Eutrema salsugineum*, a Brassicaceae plant that experienced the β and α WGDs), *A. lyrata* (a Brassicaceae plant that experienced the β and α WGDs), and *A. Ahalleri* (a Brassicaceae plant that experienced the β and α WGDs) [40,41,42,43,44,45,46,47]. In accordance with previous studies [18,30], two members that include one H-form and one L-form were identified from both updated genomes of both *A. trichopoda* and castor bean [42,44]. By contrast, three to seven members were found in other species (Table S2). Interestingly, though the majority of *caleosin* genes identified in this study possess five introns with the conserved phase pattern of 1, 1, 0, 2, and 2, *RcCLO2* was shown to contain six introns with the phases of 1, 1, 0, 2, 2, and 2, as observed in *CpCLO3* (Table S2), implying independent gain of such an intron.

**Figure 3:**
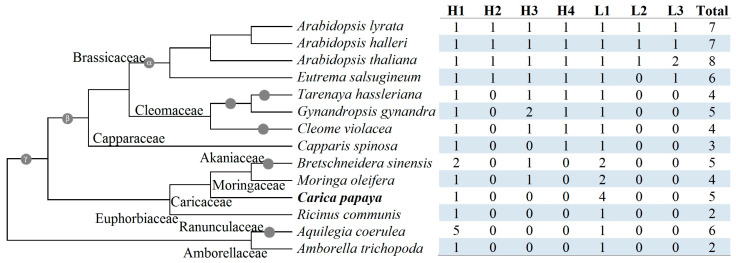
Species-specific distribution of seven caleosin orthogroups in 14 representative plant species. The species tree refers to NCBI Taxonomy (https://www.ncbi.nlm.nih.gov/taxonomy, accessed on 31 January 2025) and well-established recent WGDs are marked, which include the γ WGT shared by all core eudicots, the β WGD shared by the majority of Brassicales plants, and the α WGD specific to Brassicaceae. Names of tested plant families are indicated next to the corresponding branches. (H: high molecular weight; L: low molecular weight).

To infer lineage-specific evolution, orthogroups were identified using Orthofinder. As shown in Figure 3, a total of seven orthogroups were obtained. Among them, H1 and L1 are widely present, including *A. trichopoda* and castor bean, whereas others appear to be lineage-specific. Unlike papaya and caperbush, all other Brassicales species examined in this study contain at least one H3 member, whereas species sharing the β WGD possess one H4 member. Moreover, all tested Brassicaceae species harbor at least one L3 member, whereas three *Arabidopsis* plants contain one L2 member (Figure 3). Notably, compared with other H4 members, the short peptide length of AtCLO8 was shown to result from a “T” base in the fourth exon (Figure S2). Moreover, compared with *A. thaliana*, both *A. lyrata* and *A. Ahalleri* only retain gene fragments homologous to *AtCLO7*, implying *Arabidopsis*-specific tandem duplication followed by pseudogenization in these two species.

**Figure 4:**
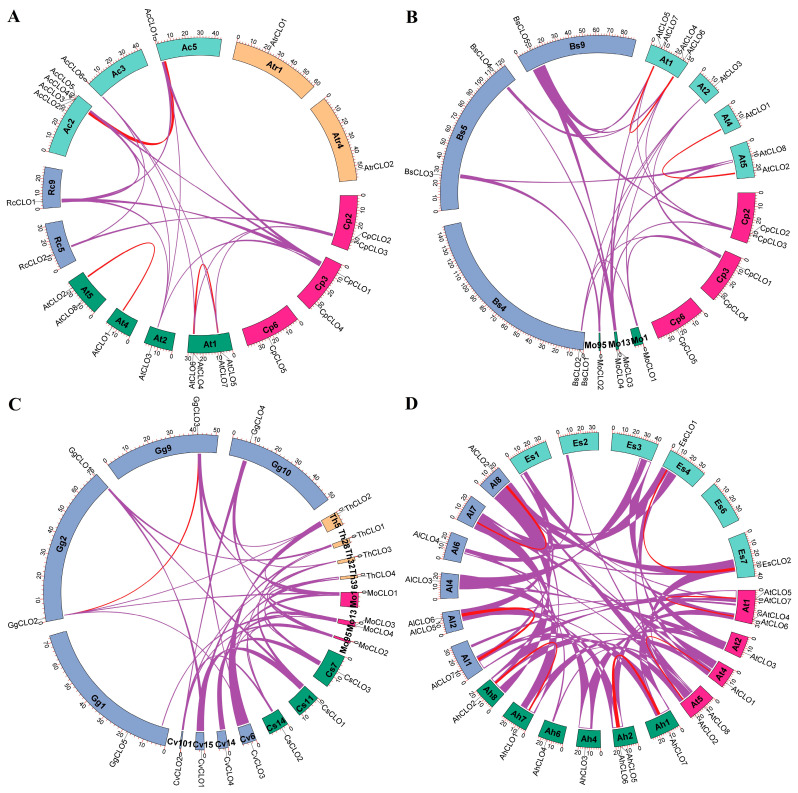
Synteny analyses within and between *C. papaya* and representative plant species. (**A**) Synteny analysis within and between *C. papaya*, *A. thaliana*, *R. communis*, *A. coerulea*, and *A. trichopoda*. (**B**) Synteny analysis within and between *C. papaya*, *M. oleifera*, *B. sinensis*, and *A. thaliana*. (**C**) Synteny analysis within and between *M. oleifera*, *C. spinosa*, *C. violacea*, *G. gynandra*, and *T. hassleriana*. (**D**) Synteny analysis within and between *A. thaliana*, *A. halleri*, *A. lyrata*, and *E. salsugineum*. Syntenic blocks were inferred using MCScanX (v2.0) (*E*-value ≤ 1 × 10^−10^; BLAST hits ≥ 5), and the circos diagram was conducted using TBtools-II. Shown are *caleosin* gene-encoding chromosomes/scaffolds and only syntenic blocks containing *caleosin* genes are marked, where red and purple lines indicate intra- and inter-species, respectively. The scale is in Mb. (Ac: *A. coerulea*; Ah: *A. halleri*; Al: *A. lyrata*; At: *A. thaliana*; Atr: *A. trichopoda*; Bs: *B. sinensis*; CLO: caleosin; Cp: *C. papaya*; Cs: *C. spinosa*; Cv: *C. violacea*; Es: *E. salsugineum*; Gg: *G. gynandra*; Mo: *M. oleifera*; Rc: *R. communis*; Th: *T. hassleriana*).

To learn more about the origin and evolution of *caleosin* genes, species-specific duplication events and interspecific syntenic analyses were further conducted. Notably, the H clade has extensively expanded in *A. coerulea* via WGD, proximal, and tandem duplications (Table S2), resulting in five members in H1, in stark contrast to species-specific expansion of the L1 group in papaya (four members) (Figure 3). Similarly to papaya, no WGD repeat was found in *A. trichopoda*, castor bean, horseradish tree, caperbush, *C. violacea*, or spider flower. By contrast, one to two WGD repeats were identified in other species, i.e., *A. coerulea* (*AcCLO1*/–*2*), *B. sinensis* (*BsCLO1*/–*3* and *BsCLO4*/–*5*), acaya (*GgCLO2*/–*3*), saltwater cress (*EsCLO1*/–*2* and *EsCLO5*/–*6*), *A. lyrata* (*AlCLO1*/–*2* and *AlCLO5*/–*7*), and *A. Ahalleri* (*AhCLO1*/–*2* and *AhCLO5*/–*7*) (Table S2), reflecting the occurrence of one or more recent WGDs [42,46,47]. Interestingly, though *AtrCLO2* was characterized as a transposed repeat of *AtrCLO1*, L1 (e.g., *RcCLO2*) was usually characterized as a dispersed repeat of H1 (e.g., *RcCLO1*) (Table S2). Since no syntelog was identified for either *AtrCLO1* or –*2* in all other species examined in this study, species-specific chromosome rearrangement or translocation in *A. trichopoda* could be speculated. By contrast, two out of five *CpCLO* genes, i.e., *CpCLO1* and –*2*, were shown to have syntelogs in most tested species, with the exception of *A. trichopoda*, implying a conserved evolution of H1 and L1. Notably, though *AtCLO3* was characterized as a dispersed repeat of *AtCLO1* residing in H3, it was shown to be located within syntenic blocks with *CpCLO1*, *RcCLO1*, *AcCLO1*, and *AcCLO2* (Figure 4A), implying WGD-derivation of H3 from H1 followed by chromosome rearrangement or translocation. Moreover, despite the fragmented status of the horseradish tree genome in 33,332 scaffolds, besides *MoCLO1* (H1) and –*3* (L1), *MoCLO2* (H3) also harbors syntelogs in *B. sinensis* (*BsCLO3*), *A. thaliana* (*AtCLO3* and –*8*) (Figure 4B), caperbush (*CsCLO2*), *C. violacea* (*CvCLO2* and –*3*), acaya (*GgCLO4*), spider flower (*ThCLO2*), *A. coerulea* (*AcCLO1* and –*2*) (Figure 4C), castor bean (*RcCLO1*), and *A. coerulea* (*AcCLO1* and –*2*) (Figure S3), implying early origin of H3. Since horseradish tree and castor bean did not undergo any recent WGD after the γ WGT shared by all core eudicots [34], the syntenic relationships of 2:1 and 2:2 observed between horseradish tree (*MoCLO1* and –*2*) and castor bean (*RcCLO1*)/*A. coerulea* (*AcCLO1* and –*2*) (Figure S3) imply the birth of H3 from the γ WGT, followed by species-specific gene loss in papaya, caperbush, and castor bean (Figure 3). The location of *MoCLO2* (H3), *CvCLO2* (H3), *CvCLO3* (H4), *AtCLO3* (H3), and *AtCLO8* (H4) within syntenic blocks (Figure 4B,C) provides direct evidence of H4 from H3, most likely as a result of the β WGD, though transposition was observed in four Brassicaceae plants (Table S2). By contrast, H2 and L3, which are specific to Brassicaceae plants and are all located within syntenic blocks with H1 and L1, respectively (Figure 4D), are most likely to arise from the α WGD. Moreover, the majority of *caleosin* genes present in four tested Brassicaceae plants are still located within syntenic blocks, exhibiting 1:1 and 2:2 relationships (Figure 4D), which is in accordance with the relatively short time of their divergence.

### 2.4. Caleosin Genes in Castor Bean and Papaya Underwent Apparent Expression Divergence

Species-specific expansion of the *caleosin* family in papaya propelled us to study their expression profiles and possible function divergence. For the purposes, expression profiles of *RcCLO* genes (each for two clades) were first examined in leaves, male flowers, II/III endosperms, V/VI endosperms, and germinating seeds. As shown in Figure S4A, similar to *RcOLE* genes, *RcCLO1* is preferentially expressed in endosperms. By contrast, *RcCLO2* is predominantly expressed in male flowers, germinating seeds, and leaves (Figure S4A), implying expression divergence of H and L forms. Notably, the total transcripts of the *RcCLO* family in male flowers and leaves are relatively more than those of the *RcOLE* family, but considerably less than those in endosperms and germinating seeds (Figure S4A).

**Figure 5:**
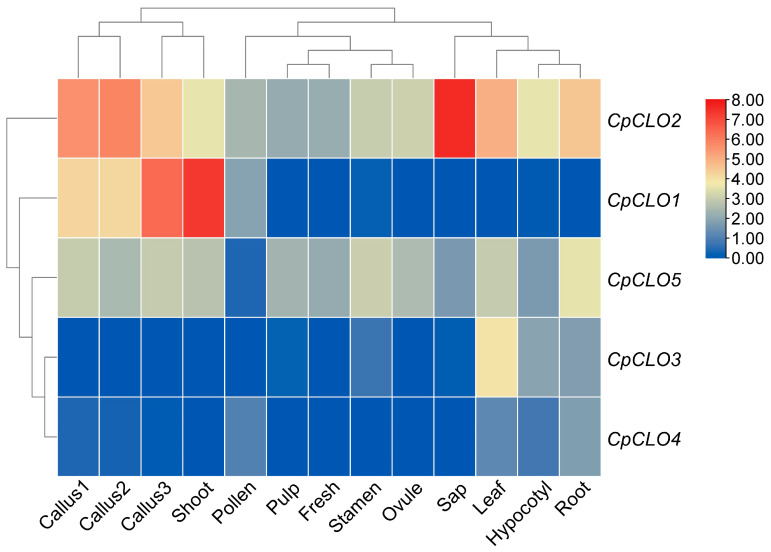
Expression profiles of *CpCLO* genes. The heatmap was generated using the R package (v3.5.0), where color scales represent FPKM normalized Log_2_ transformed counts. Blue and red indicate low and high expression, respectively. (CLO: caleosin; Cp: *C. papaya*; FPKM: fragments per kilobase of exon per million fragments mapped).

Tissue-specific expression profiles of five *CpCLO* genes are shown in Figure 5. Though their transcripts were detected in at least one of 13 samples examined in this study, i.e., callus (three stages termed I, II, and III), shoot, hypocotyl, root, leaf, sap, stamen, pollen, ovule, fresh, and pulp, distinct expression patterns were observed. Similarly to the *CpOLE* family, total transcripts of the *CpCLO* family are highly abundant in shoot and three stages of callus, though the most expressed tissue is the sap (Figure S4B). Interestingly, the majority of *CpCLO* transcripts in the sap are contributed by *CpCLO2*, a constitutively expressed member occupying 43.48–71.85% of total transcripts in pulp, stamen, fresh, pollen, leaf, root, ovule, hypocotyl, callus1, and callus2. By contrast, *CpCLO1* contributes to 74.69% and 90.34% of total transcripts in callus3 and shoot samples, respectively. *CpCLO5* is also constitutively expressed, though its abundances in most tested samples are considerably lower than those of *CpCLO2*. The expression of *CpCLO2*–*4* was shown to be tissue-specific. *CpCLO2* is highly abundant in shoot, callus3, callus1, and callus2 and has low abundance in pollen, but is rarely expressed in other tissues. *CpCLO3* is predominantly expressed in leaf, hypocotyl, and root, but rarely in other tissues, whereas *CpCLO4* is preferentially expressed in leaf, root, and pollen, but rarely in other tissues (Figure 5). Correspondingly, the shoot tissue is clustered with three stages of callus with high abundances of *CpOLE* and *CpCLO* transcripts. Moreover, *CpCLO1* is clustered with *CpOLE2*–*5*, which are highly abundant in shoot and three stages of callus; *CpCLO2* is clustered with *CpOLE6*, the unique *CpOLE* member that is constitutively expressed in all tested tissues; and *CpCLO3*–*5* are clustered with *CpOLE1* (Figure S4B). These results imply apparent expression divergence of *CpCLO* genes even between four L forms (i.e., *CpOLE2*–*5*). Additionally, similar to those observed in castor bean, the total transcripts of the *CpCLO* family in leaves, roots, and sap are relatively larger than those of the *CpOLE* family (Figure S4B).

## 3. Discussion

LDs are lipid storage compartments that are enclosed by a monolayer of phospholipids and several structural proteins such as oleosins, caleosins, and steroleosins [17]. Among them, oleosins and caleosins are typical for the presence of a 12-residue proline-knot motif, which is necessary for budding-LDs to enter the cytosol [48]. In oilseeds, the LD size is largely determined by the amounts of oleosins and caleosins [25,49]. Oleosins first appeared in the single-celled algae and have diverged into at least seven clades known as P (primitive), U (universal), SL (seed low MW), SH (seed high MW), T (tapetum), M (mesocarp), and N (novel) during later evolution [23,50]. By contrast, only two clades of caleosins known as H and L have been described in higher plants [19]. In papaya, six *oleosin* genes representing five clades (i.e., U, SL, SH, M, and N) were identified, and the comparison with representative plant species revealed that the T clade is limited to the Brassicaceae family, appearing sometime after the split with Cleomaceae [23]. To fill the gap of research on *CpCLO* genes, in this study, we first conducted a genome-wide analysis of the *caleosin* family in papaya, and further addressed lineage-specific family evolution in the whole Brassicales order.

In contrast to only two members representing two clades that were found in castor bean and *A. trichopoda* ([18,30], this study), an unexpectedly high number of five *caleosin* genes were identified in papaya, though the family amounts were lower than the 7 to 13 reported in *A. thaliana* and other Brassicales plants [19,20]. Phylogenetic analysis and sequence comparison revealed that, like *A. thaliana*, papaya also includes two clades, i.e., one H-form and four L-forms, where the H clade features an extra N-terminal insertion of 29 residues that has been named H insertion [19]. Notably, the composition is different from *A. thaliana*, which contains four H-forms and four L-forms [19], implying species- or lineage-specific expansion.

To gain insights into the origin and evolution of *caleosin* genes, a total of 63 members were further identified from 13 representative plant species, which belong to eight plant families, i.e., Amborellaceae (*A. trichopoda*), Ranunculaceae (*A. coerulea*), Euphorbiaceae (castor bean), Moringaceae (horseradish tree), Akaniaceae (*B. sinensis*), Capparaceae (caperbush), Cleomaceae (*C. violacea*, acaya, and spider flower), and Brassicaceae (saltwater cress, *A. Ahalleri*, *A. lyrata*, and *A. thaliana*). Among them, castor bean, *A. coerulea*, and *A. trichopoda* were adopted as out-groups of Brassicales plants, core eudicots, and core angiosperms, respectively. Interestingly, despite the occurrence of one or more additional WGDs after the split with papaya, comparative or a relatively smaller number of *caleosin* family genes were found in caperbush (3), *C. violacea* (4), spider flower (4), *B. sinensis* (5), and acaya (5). Moreover, only four members were identified in horseradish tree, another Brassicales plant without a recent WGD [41]. Nevertheless, four *MoCLO* genes belong to three out of the seven orthogroups identified in this study, i.e., H1, H3, and L1, in contrast with only two (i.e., H1 and L1) for five *CpCLO* genes. Since H1 and L1 were shown to be shared by *A. trichopoda*, their early origin before angiosperm radiation could be speculated. Though H3 is absent from castor bean, papaya, and caperbush, it is present in all other Brassicales plants examined in this study. An interesting result is that *RcCLO1* (H1), *MoCLO1* (H1), and *MoCLO2* (H3) are located within syntenic blocks, supporting WGD-derivation of H3 from H1, probably the γ WGT occurred in the last common ancestor of core eudicots after the split with *A. coerulea* [34,42]. Compared with papaya and horseradish tree, other tested Brassicales plants were proven to have experienced at least one recent WGD, which resulted in several duplicate pairs, i.e., one in *B. sinensis* (Bs-α), one in acaya (Gg-α), and two in four Brassicaceae plants (α). Two WGD repeats shared by saltwater cress, *A. Ahalleri*, *A. lyrata*, and *A. thaliana* formed two Brassicaceae-specific groups, i.e., H2 and L3, as defined in this study. Notably, though H4 was characterized as the transposed repeat of H3 in all four tested Brassicaceae plants, they were all shown to be located within syntenic blocks with the H3 member in both horseradish tree (*MoCLO2*) and *C. violacea* (*CvCLO2*), supporting WGD-derivation of H4 from H3—probably the β WGD shared by several families within Brassicales, e.g., Capparaceae, Cleomaceae, and Brassicaceae [45,47]. Additionally, since this transposed event is not found in other species beyond Brassicaceae, its appearance in the last common ancestor of Brassicaceae plants could be speculated. Taken together, possible evolution routes of the *caleosin* family in Brassicales are as follows: the family first diverged into H1 and L1 before angiosperm radiation, and the γ WGT gave birth to H3, though it has been lost in castor bean, papaya, and caperbush; in papaya, the L clade underwent extensive expansion via species-specific tandem and transposed duplications; in horseradish tree, the L clade also underwent species-specific expansion via tandem duplication; in *B. sinensis*, H1 and L1 underwent species-specific expansion via tandem duplication and Bs-α WGD, respectively; the β WGD gave rise to H4 from H3, though H3 was lost in caperbush during latter evolution; in acaya, H3 was further expanded via the Gg-α; in Brassicaceae, the α WGD resulted in H2 and L3, whereas L1 in *Arabidopsis* further gave birth to L2 via tandem duplication; L3 was also expanded via tandem duplication, which was retained in *A. thaliana* but underwent pseudogenization in both *A. lyrata* and *A. Ahalleri*.

In addition to local duplication and species-specific retention of repeats after WGDs, structural and expression divergences were also shown to play key roles in the evolution of the *caleosin* family. Generally, *caleosin* genes feature five introns in the phase pattern of 1, 1, 0, 2, and 2. However, independent gain or loss of certain introns was also observed in *CpCLO3*, *RcCLO2*, *AtCLO8*, and *AtCLO7*. Moreover, unusual intron phases of 2, 0, 0, 0, 2, and 2 were also observed in *AtCLO7*. Lineage or species-specific gain of certain introns was also reported for the *oleosin* family, e.g., *CpOLE3* [20,21,23,48], though their biological significances have not been clarified.

Over more than 200 million years of evolution, apparent expression divergence was observed between two clades of *caleosin* genes. A good example is castor bean, an oil seed crop of the Euphorbiaceae family [44] possessing a single H-form and one L-form. Whereas the L-form *RcCLO2* is constitutively expressed, the H-form *RcCLO1* has evolved to be preferentially expressed in seeds/endosperms, which are consistent with a previous study [18]. Though no seed transcriptome is available in papaya, analyzing 13 tissue samples showed that the H-form *CpCLO1* is predominantly expressed in shoots and the last stage of callus development, when *CpOLE* genes are highly abundant [23]. As for four L-forms, *CpCLO2* and –*5* were shown to be constitutively expressed, whereas *CpCLO3* and –*4* exhibit apparent tissue-specific expression patterns. Moreover, *CpCLO2* has evolved into the dominant member, whose transcripts are usually more than other three L-forms in most tissues, implying its putatively key roles. In *A. thaliana*, the situation is more complex, as extensive expansion was observed in both clades, i.e., four H-forms and four L-forms [19]. Among the four H-forms, *AtCLO1* and –*2* were shown to exhibit the seed-preferential expression pattern similar to *RcCLO1*, whereas *AtCLO3* appears to be constitutively expressed [19,51]. Among the four L-forms, *AtCLO4* is also constitutively expressed, whereas the other three members seem to be predominantly expressed in flowers [19]. Moreover, the involvement of *AtCLO1*, –*2*, –*4*, and –*6* in oil accumulation and embryo development was supported by mutant analyses, which were shown to have overlapping functions [51]. *AtCLO1* was also shown to be involved in the degradation of storage lipids during seed germination [3], whereas *AtCLO2* was proven to be associated with seed dormancy [52]. The PXG activity, which is Ca^2+^-dependent, has been reported for AtCLO1–4 [7,24,53]. *AtCLO3* was functionally characterized in the generation of oxidized fatty acids in the stress-related ABA and salicylic acid signaling pathways [7], whereas *AtCLO4* was characterized as a negative regulator of the ABA response [53].

## 4. Conclusions

This study presents the first genome-wide analysis of the *caleosin* family in papaya, resulting in a high number of five members representing two clades, i.e., H (1) and L (4). Comparison of 68 *caleosin* genes from 14 representative plant species revealed seven orthogroups, i.e., H1–4 and L1–3, two of which (i.e., H1 and L1) have appeared before angiosperm radiation. Five *CpCLO* genes belong to H1 and L1, and extensive expansion of the L1 group appears to be contributed by species-specific tandem and transposed duplications, which may contribute to environmental adaptation. By contrast, WGDs were shown to play a more important role in family expansion in Brassicaceae plants such as *A. thaliana*. Structural (e.g., gain or loss of certain introns) and apparent expression divergence of *caleosin* genes was also observed. These findings provide a global view of *CpCLO* genes and insights into lineage-specific family evolution in Brassicales, which facilitates further functional studies in papaya and other nonmodel species.

## 5. Materials and Methods

### 5.1. Databases and Identification of Caleosin Family Genes

Genomic sequences of representative plant species, i.e., *A. trichopoda* (v2.1; Amborellaceae, Amborellales), *A. coerulea* (v3.1; Ranunculaceae, Ranunculales), *R. communis* (WT05; Euphorbiaceae, Malpighiales), *C. papaya* (Sunset v1; Caricaceae, Brassicales), *M. oleifera* (v1; Moringaceae, Brassicales), *B. sinensis* (v1; Akaniaceae, Brassicales), *C. spinosa* (v1; Capparaceae, Brassicales), *C. violacea* (v2.1; Cleomaceae, Brassicales), *G. gynandra* (v1; Cleomaceae, Brassicales), *T. hassleriana* (v1; Cleomaceae, Brassicales), *E. salsugineum* (v1.0; Brassicaceae, Brassicales), *A. halleri* (v2.1; Brassicaceae, Brassicales), *A. lyrata* (v2.1; Brassicaceae, Brassicales), and *A. thaliana* (Araport11; Brassicaceae, Brassicales), were downloaded from NGDC (http://bigd.big.ac.cn/gsa, accessed on 31 January 2025), Phytozome (v13, https://phytozome.jgi.doe.gov/pz/portal.html, accessed on 31 January 2025), and NCBI (https://www.ncbi.nlm.nih.gov/, accessed on 31 January 2025). The caleosin domain profile (PF05042) was obtained from Pfam 33.1 (https://pfam.xfam.org/, accessed on 31 January 2025), which was used for HMMER (v3.3, http://hmmer.janelia.org/, accessed on 31 January 2025) searches, as described before [54]. Gene models of candidates were manually curated with available mRNAs, and presence of the conserved caleosin domain in deduced peptides was confirmed using MOTIF Search (https://www.genome.jp/tools/motif/, accessed on 31 January 2025). Pseudogenes and/or homologous fragments were identified with coding sequences (CDSs) of obtained *caleosin* genes using BLASTN (v2.17.0) as previously described [55]. Exon–intron structures were displayed using GSDS2.0 (http://gsds.cbi.pku.edu.cn/, accessed on 31 January 2025), whereas protein properties, hydropathicity scales, and secondary structures were determined using ProtParam (http://web.expasy.org/protparam/, accessed on 31 January 2025), ProtScale (v1) (https://web.expasy.org/protscale/, accessed on 31 January 2025), and SOPMA (v1) (https://npsa-prabi.ibcp.fr/cgi-bin/npsa_automat.pl?page=/NPSA/npsa_sopma.html, accessed on 31 January 2025), respectively.

### 5.2. Multiple Sequence Alignment, Phylogenetic, and Conserved Motif Analyses

Sequence alignment of nucleotides and proteins was performed using ClustalW (v2.1, www.megasoftware.net, accessed on 31 January 2025) and MUSCLE (v5, http://www.drive5.com/muscle/, accessed on 31 January 2025), respectively, and alignment display was carried out using Boxshade (v1) (https://embnet.vital-it.ch/software/http://www.bio-soft.net/sms/, accessed on 31 January 2025). A phylogenetic tree was constructed using RAxML (v2) (http://www.phylo.org/portal2/home.action#, accessed on 31 January 2025) as previously described [56], which implemented the maximum likelihood method and bootstrap of 1000 replicates. Conserved motifs were identified using MEME (v5.4.1, http://meme-suite.org/tools/meme, accessed on 31 January 2025) with the following parameters: any number of repetitions; the maximum number of motifs, 10; and the optimum width of each motif, between 5 and 200 residues.

### 5.3. Definition of Orthogroups, Chromosomal Localization, Synteny Analysis, and Calculation of Evolutionary Rate

Orthologous genes were clustered using Orthofinder (v2.3.8) [57], whereas chromosomal localization was conducted using TBtools-II (v2.210) [58]. For synteny analysis, duplicate pairs were identified using the all-to-all BLASTp (v2.17.0) method with the *E*-value cutoff of 1 × 10^−10^, and gene colinearity was inferred using MCScanX (v2.0) with the cutoff of five BLAST hits as previously described [56]. Duplication modes such as tandem, proximal, transposed, dispersed, and WGD were identified using the DupGen_finder pipeline [59], and the Ks and Ka of duplicate pairs were calculated using codeml [60].

### 5.4. Gene Expression Analysis

Global expression profiles of *RcCLO* and *CpCLO* genes were analyzed on the basis of Illumina RNA-seq samples, as shown in Table S3. Five samples examined in castor been are the cotyledons of germinating seeds, expanding true leaves that appear after the first cotyledons and leaf-pair, developing male flowers, endosperm II/III (free-nuclear stage of developing seeds), and endosperm V/VI (onset of cellular endosperm development). The thirteen samples tested in papaya are hypocotyl (7 days on MS medium), callus I (21 days on callus induction medium K5), callus II (21 days on callus induction medium M13), callus III (21 days on callus induction medium CI), shoot (28 days on shoot induction medium), root, leaf, phloem sap, stamen, pollen, and ovule. Quality control of raw reads was carried out using Trimmomatic (v1) [61], and read mapping was performed using HISAT2 [62]. Relative gene expression levels were normalized using the FPKM (fragments per kilobase of exon per million fragments mapped) method [63], whereas differential analysis was conducted using DESeq2 (v1.22.1) [64] under the criteria of |log_2_ fold change (FC)| ≥ 1 and false discovery rate (FDR) < 0.05. Unless specifically stated, the tools in this study were used with default parameters.

## Figures and Tables

**Table 1:** *Caleosin* genes identified in *C. papaya*. (AA: amino acid; Chr: chromosome; CLO: caleosin; Cp: *C. papaya*; GRAVY: grand average of hydropathicity; kDa: kilodalton; MW: molecular weight; pI: isoelectric point).

Gene Name	Locus	Position	AA	MW (kDa)	pI	GRAVY	Caleosin Location	Clade
*CpCLO1*	sunset03G0007900	Chr3:6306411..6308488(+)	240	27.24	5.05	−0.329	62..228	H
*CpCLO2*	sunset02G0020020	Chr2:31227224..31229504(+)	204	22.67	6.09	−0.419	25..192	L
*CpCLO3*	sunset02G0020030	Chr2:31241163..31242710(+)	206	23.57	6.80	−0.402	25..192	L
*CpCLO4*	sunset03G0022160	Chr3:31772679..31773742(+)	208	23.89	9.41	−0.401	27..194	L
*CpCLO5*	sunset06G0015560	Chr6:23171674..23173752(+)	205	23.67	9.57	−0.509	26..192	L

**Table 2:** *Caleosin* duplicates identified in *C. papaya*. Ks and Ka were calculated using PAML. (CLO: caleosin; Cp: *C. papaya*; Ka: nonsynonymous substitution rate; Ks: synonymous substitution rate).

Gene1	Gene2	Identity (%)	Ka	Ks	Ka/Ks
*CpCLO1*	*CpCLO2*	46.8	0.5152	-	-
*CpCLO2*	*CpCLO3*	71.2	0.2378	1.2020	0.1978
*CpCLO2*	*CpCLO4*	67.5	0.2646	1.2871	0.2056
*CpCLO2*	*CpCLO5*	69.6	0.2757	0.8983	0.3069

## Data Availability

The datasets analyzed during the current study are available in the NCBI SRA repository (https://www.ncbi.nlm.nih.gov/sra/ (accessed on 31 January 2025)), whose accession numbers are shown in Table S3.

## Supplementary Materials

The following supporting information can be downloaded at https://www.mdpi.com/article/10.3390/plants14213296/s1, Figure S1: Chromosome localization and duplication events detected in *A. thaliana*. Chromosome localization was conducted using TBtools-II, whereas different modes of gene duplication were identified using the DupGen_finder pipeline. Serial numbers are indicated at the top of each chromosome, and the scale is in Mb. Duplicate pairs identified in this study are connected using lines in different colors, i.e., WGD (red), tandem (green), transposed (purple), and dispersed (gold). (At: *A. thaliana*; CLO: caleosin). Figure S2: Sequence alignment of *AlCLO4*, *AhCLO4*, and *AtCLO8*. Sequence alignment was performed using ClustalX. Broken lines in the sequences represent gaps introduced for best alignment, whereas identical nucleotides are indicated using asterisks. (Ah: *A. halleri*; Al: *A. lyrata*; At: *A. thaliana*; CLO: caleosin). Figure S3: Synteny analyses of *caleosin* genes within and between *M. oleifera*, *R. communis*, and *A. coerulea*. Syntenic blocks were inferred using MCScanX (*E*-value ≤ 1 × 10^−10^; BLAST hits ≥ 5). Shown are *caleosin* gene-encoding chromosomes/scaffolds and only syntenic blocks containing *caleosin* genes are marked, where red and purple lines indicate intra- and inter-species, respectively. (Ac: *A. coerulea*; CLO: caleosin; Mo: *M. oleifera;* Rc: *communis*). Figure S4: Expression profiles of *caleosin* and *oleosin* genes in *R. communis* (A) and *C. papaya* (B). The heatmaps were generated using the R package, where color scales represent FPKM normalized Log_2_ transformed counts. Blue and red indicate low and high expression, respectively. (CLO: caleosin; Cp: *C. papaya*; FPKM: fragments per kilobase of exon per million fragments mapped). Table S1: Percent similarity within and between CpCLOs and AtCLOs. (At: *A. thaliana*; CLO: caleosin). Table S2: List of *caleosin* genes identified in *A. thaliana* and representative plant species. (Ac: *A. coerulea*; Ah: *A. halleri*; Al: *A. lyrata*; At: *A. thaliana*; Atr: *A. trichopoda*; Bs: *B. sinensis*; CLO: caleosin; Cs: *C. spinosa*; Cv: *C. violacea*; Es: *E. salsugineum*; Gg: *G. gynandra*; Mo: *M. oleifera*; Rc: *R. communis*; Th: *T. hassleriana*). Table S3: Detailed information of transcriptome data used in this study.
