# Supplementary figures and images for: Characterization of Caleosin Genes in Carica papaya and Insights into Lineage-Specific Family Evolution in Brassicales
Source: Plants (Basel). 2025 Oct 29;14(21):3296. doi: 10.3390/plants14213296 (PMC12610490; doi:10.3390/plants14213296)

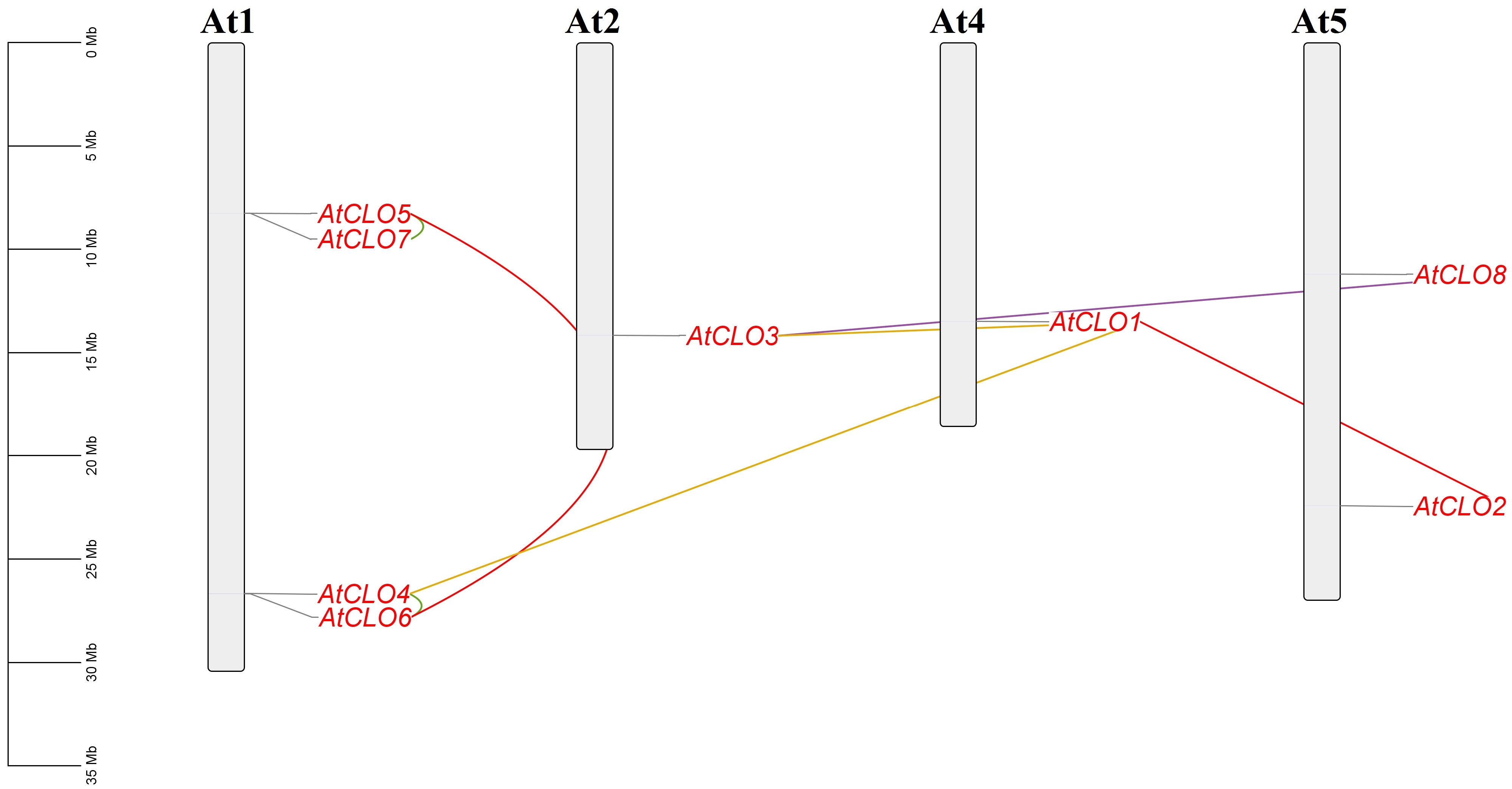

Supplement: Supplementary file 1 [file plants-14-03296-s001.zip › Figure S1.jpg]

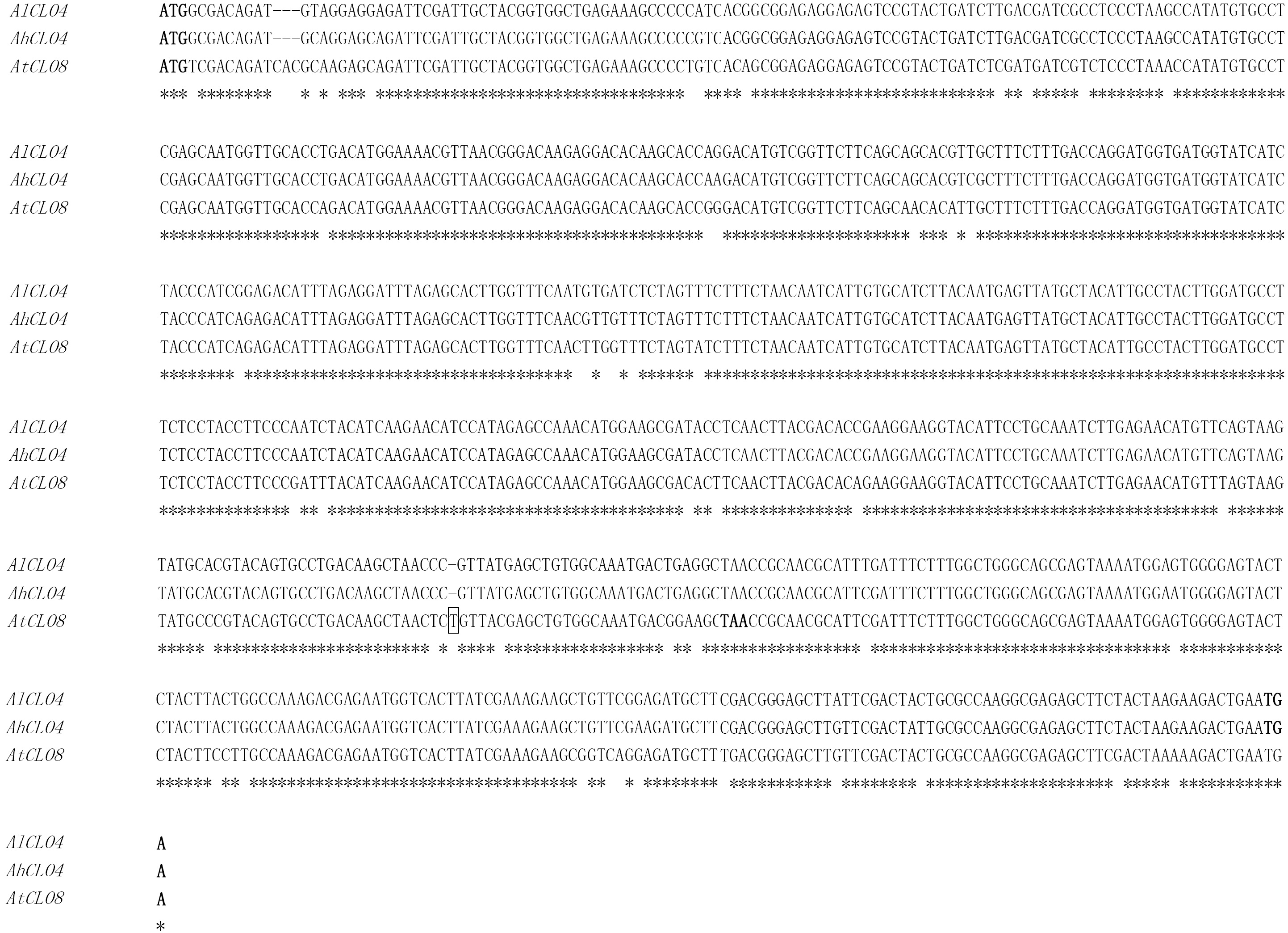

Supplement: Supplementary file 1 [file plants-14-03296-s001.zip › Figure S2.jpg]

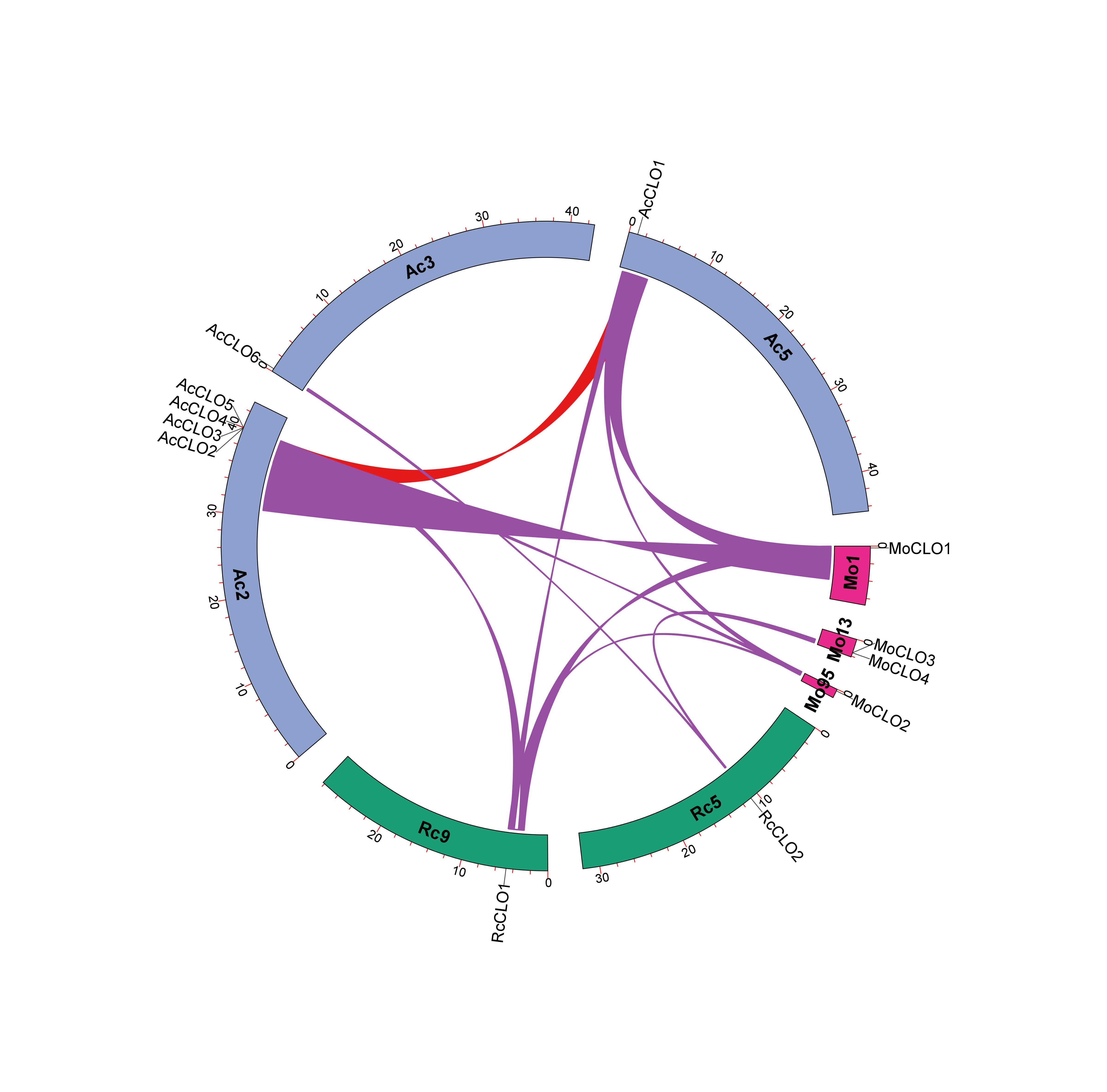

Supplement: Supplementary file 1 [file plants-14-03296-s001.zip › Figure S3.jpg]

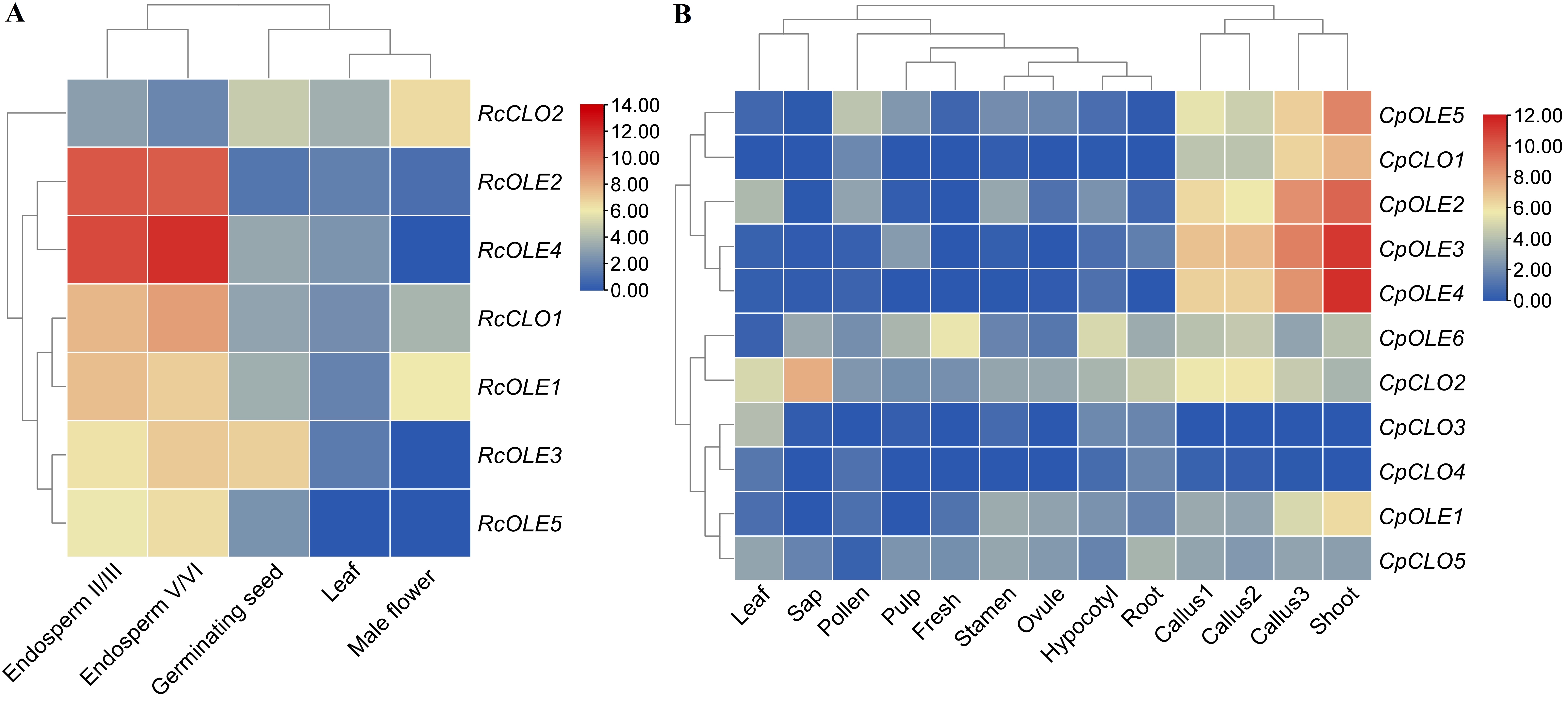

Supplement: Supplementary file 1 [file plants-14-03296-s001.zip › Figure S4.jpg]
